# An Impairment in Resting and Exertional Breathing Pattern May Occur in Long-COVID Patients with Normal Spirometry and Unexplained Dyspnoea

**DOI:** 10.3390/jcm11247388

**Published:** 2022-12-13

**Authors:** Annalisa Frizzelli, Francesco Di Spigno, Luca Moderato, Geza Halasz, Marina Aiello, Panagiota Tzani, Gaia Manari, Luigino Calzetta, Roberta Pisi, Giovanna Pelà, Massimo Piepoli, Alfredo Chetta

**Affiliations:** 1 Department of Medicine and Surgery, University of Parma, 43126 Parma, Italy; 2 Cardio-Thoracic and Vascular Department, University Hospital of Parma, 43126 Parma, Italy; 3 Cardiac Unit, “G. da Saliceto” Hospital of Piacenza, 29121 Piacenza, Italy; 4 Department of General and Specialistic Medicine, University Hospital of Parma, 43126 Parma, Italy; 5 Clinical Cardiology Unit, Policlinico San Donato, University of Milan, 20122 Milan, Italy; 6 Department of Preventive Cardiology, Wroclaw Medical University, 50-367 Wroclaw, Poland

**Keywords:** COVID-19, spirometry, dyspnoea

## Abstract

**Background:** Long-term sequelae, called Long-COVID (LC), may occur after SARS-CoV-2 infection, with unexplained dyspnoea as the most common symptom. The breathing pattern (BP) analysis, by means of the ratio of the inspiratory time (T_I_) during the tidal volume (V_T_) to the total breath duration (T_I_/T_TOT_) and by the V_T_/T_I_ ratio, could further elucidate the underlying mechanisms of the unexplained dyspnoea in LC patients. Therefore, we analysed T_I_/T_TOT_ and V_T_/T_I_ at rest and during maximal exercise in LC patients with unexplained dyspnoea, compared to a control group. **Methods:** In this cross-sectional study, we enrolled LC patients with normal spirometry, who were required to perform a cardio-pulmonary exercise test (CPET) for unexplained dyspnoea, lasting at least 3 months after SARS-CoV-2 infection. As a control group, we recruited healthy age and sex-matched subjects (HS). All subjects performed spirometry and CPET, according to standardized procedures. **Results:** We found that 42 LC patients (23 females) had lower maximal exercise capacity, both in terms of maximal O_2_ uptake (VO_2_peak) and workload, compared to 40 HS (22 females) (*p* < 0.05). LC patients also showed significantly higher values of T_I_/T_TOT_ at rest and at peak, and lower values in V_T_/T_I_ at peak (*p* < 0.05). In LC patients, values of T_I_/T_TOT_ at peak were significantly related to ∆PETCO_2_, i.e., the end-tidal pressure of CO_2_ at peak minus the one at rest (*p* < 0.05). When LC patients were categorized by the T_I_/T_TOT_ 0.38 cut-off value, patients with T_I_/T_TOT_ > 0.38 showed lower values in VO_2_peak and maximal workload, and greater values in the ventilation/CO_2_ linear relationship slope than patients with T_I_/T_TOT_ ≤ 0.38 (*p* < 0.05). **Conclusions:** Our findings show that LC patients with unexplained dyspnoea have resting and exertional BP more prone to diaphragmatic fatigue, and less effective than controls. Pulmonary rehabilitation might be useful to revert this unpleasant condition.

## 1. Introduction

After the resolution of the acute phase, SARS-CoV-2 infection may present important clinical-functional long-term sequelae. The term “Long-COVID” (LC) includes both “persistent symptomatic COVID-disease” and “post-COVID-19 syndrome”. The former considers signs and symptoms related to SARS-CoV-2 lasting between 4 and 12 weeks after the acute phase; the latter refers to signs and symptoms compatible with COVID-19, present for more than 12 weeks after the acute phase, without alternative aetiologies [1]. Respiratory symptoms are the main symptoms during LC, with dyspnoea and fatigue on exertion being the most common complaints [2].

Unexplained dyspnoea is one of the main indications for the cardiopulmonary exercise test (CPET) and several reports have been recently published on the CPET profiles of LC patients [3,4,5,6,7,8]. The first relevant studies in LC patients with unexplained dyspnoea reported a CPET profile of deconditioning, because of an acute inflammatory process, prolonged bed rest, and post-traumatic syndrome and depression [3,4,5]. Subsequently, abnormal ventilatory response to exercise with dysfunctional breathing (DB) was recognized in a range from 29% [7] to 63% [6,8] of the LC patients. Different patient selection criteria may explain the different rates of DB in LC patients, although also the lack of a gold standard to diagnose DB [9,10] might play a role.

A simple and well-known way of analysing the breathing pattern (BP) is to measure the ratio of the inspiratory time (T_I_) during the tidal volume (V_T_), to the total breath duration (T_I_/T_TOT_) as well as the inspiratory flow, i.e., the ratio of V_T_ to T_I_ [11]. Importantly, T_I_/T_TOT_ has been termed the duty cycle of the respiratory system and V_T_/T_I_ has been widely employed as a measure of respiratory drive [11]. So far, an analysis of BP during exercise has only been reported in healthy subjects [12].

We hypothesized that in LC patients with unexplained dyspnoea, the analysis of BP by means of T_I_/T_TOT_ and V_T_/T_I_ at rest and during maximal exercise could further elucidate the underlying mechanisms of the symptom. Therefore, we performed CPET in a large cohort of LC patients with normal spirometry and suffering from unexplained dyspnoea, in comparison to a control group. In all subjects, we analysed T_I_/T_TOT_ and V_T_/T_I_ along with the traditional CPET parameters.

## 2. Methods

### 2.1. Patients

In this cross-sectional study, we prospectively recruited consecutive adult patients with a previous PCR confirmed COVID-19, who were referred for CPET at the Lung Function Unit of the University Hospital of Parma, and at the Cardiac Unit of the “G. da Saliceto” Hospital of Piacenza for unexplained dyspnoea lasting at least 3 months after SARS-CoV-2 infection. We excluded patients with concomitant heart or lung disease, or with an abnormal spirometry. The study was approved by the Ethics Committee of North Emilia (approval number: 131, dated: 18 March 2022). We also enrolled healthy, age, sex and Body Mass Index (BMI, kg/m^2^)-matched subjects (HS) who had never smoked to serve as a control group; patients were recruited during the routine outpatient clinic according to 1:1 ratio.

### 2.2. Pulmonary Function and Cardiopulmonary Exercise Test

Pulmonary function tests were performed according to international recommendations [13]. A flow-sensing spirometer connected to a computer for data analysis (Vmax 22 and 6200, Sensor Medics, Yorba Linda, CA, USA) was used for the measurements. Forced vital capacity (FVC) and forced expiratory volume at 1st second (FEV_1_) were recorded and expressed as percentage of the predicted values, which were obtained from regression equations [14].

CPET was performed according to a standardised procedure [15]. After calibrating the oxygen and carbon dioxide analysers and flow mass sensor, patients were asked to sit on an electromagnetically braked cycle ergometer (Corival PB, Lobe Bv, Groningen, The Netherlands; Cosmed, Rome, Italy) and the saddle was adjusted properly to avoid the maximal extension of the knee. The exercise protocol involved an initial 3 min of rest, followed by unloaded cycling for another 3 min with an increment every minute of 5–20 Watts, according to the patient’s anthropometry, in order to achieve an exercise time in between 8 and 12 min. Patients were asked to maintain a pedalling frequency of 60 rotations/min (rpm) indicated by a digital display placed on the monitor of the ergometer.

Breath-by-breath oxygen uptake (VO_2_ in mL/kg/min), carbon dioxide production (VCO_2_ in mL/kg/min), tidal volume (V_T_ in L), respiratory rate (RR in bpm) and minute ventilation (VE in L/min) were recorded during the test (CPX/D; Med Graphics, St. Paul, MN, USA; Quark CPET, Cosmed, Rome, Italy). Patients were continuously monitored by a 12-lead electrocardiogram (Welch Allyn CardioPerfect, Delft, The Netherlands) and a pulse oximeter (Pulse Oximeter 8600, Nonin Medical Inc., MPLS, MN, USA; Cosmed, Rome, Italy). Blood pressure was measured at 2 min intervals. Exercise was stopped according to the standardised criteria [15]. Predicted values were calculated according to equations by Wasserman et al. [16].

Peak workload (in watts) and peak VO_2_ (in mL/kg/min) were recorded as the mean value of watts and VO_2_ during the last 20 s of the test. Anaerobic threshold (AT) was non-invasively determined by both V-slope and ventilatory equivalents methods (“dual method approach”) [15] and was expressed as absolute value of VO_2_ in mL/kg/min.

The breathing reserve (BR, %) was calculated by the formula 1-(peak ventilation/maximum voluntary ventilation) * 100. Maximum voluntary ventilation was obtained by multiplying FEV_1_ by 40. The ventilatory response during exercise was expressed as a linear regression function by plotting VE against VCO_2_ obtained every 10 s, excluding data above the ventilatory compensation point [15]. Then, the slope values were obtained from the VE/VCO_2_ regression line. The end-tidal pressure of CO_2_ (PETCO_2_, in mmHg) was measured as mean of PETCO_2_ during the 3 min rest period and during the last 20 s of the test and was also recorded as the difference between PETCO_2_ peak and PETCO_2_ rest (∆PETCO_2_). At rest and during exercise the pattern of breathing was assessed by recording T_I_/T_TOT_ and V_T_/T_I_.

The cardiovascular response to exercise was expressed as oxygen pulse (O_2_Pulse) and oxygen uptake efficiency slope (OUES) i.e., the relation between oxygen uptake and ventilation [17] and as the heart rate recovery at peak of exercise (HRR, in bpm) [18]. Dyspnoea induced by incremental exercise was measured at the end of the exercise by a visual analogue scale (VAS), which consisted of a 100 mm horizontal line with the word “none” placed at the left end of the scale and the words “very severe” placed at the right of the scale. The VAS scored from 0 to 100. Dyspnoea perception ratings were then divided by the maximal workload (VAS dyspnoea, in mm/watts) for analysis.

### 2.3. Statistical Analysis

This is a pilot, cross-sectional study. Due to the explorative nature of the study no formal sample size calculation was performed. Data are reported as mean ± standard deviation (SD), unless otherwise specified. The distribution of variables was assessed by means of a Kolmogorov–Smirnov goodness-of-fit test.

Relationships between variables were assessed by the Pearson’s correlation coefficient (r) and linear regression analysis or Spearman correlation coefficient (rs), when appropriate. Comparisons between variables were determined by unpaired t-test or by Chi-square test, when appropriate. The T_I_/T_TOT_ cut-off value of 0.38 was chosen a posteriori, since it was the median T_I_/T_TOT_ value at the peak of exercise in the control group.

Appropriate curve-fitting models were identified to analyse during exercise: T_I_/T_TOT_, [Y = (Y0-Plateau) * exp(-K * X) + Plateau, where Y0 is the Y value when X is zero, plateau is the Y value at infinite values, and K is the rate constant expressed in reciprocal of the X axis] and V_T_/T_I_ [Y = Y Intercept + Slope * X, where Y Intercept is the Y value where the line intersects the Y axis, and slope is the slope of the line, expressed in Y units divided by X units].

A *p* value of less than 0.05 was taken as significant. Statistical analysis and diagrams were obtained by Prism 8 (©2018 GraphPad Software, La Jolla, CA, USA).

## 3. Results

Fifty-two LC patients and forty HS controls, respectively, aged between 22 and 66 years and between 26 and 79 years, were studied. In LC patients, spirometry values were in the normal range, although FEV_1_ values were significantly lower than for HS controls (Table 1).

All subjects completed the exercise test without any complications and no subjects were excluded because of poor motivation. The average interval between onset of SARS-CoV-2 infection to CPET was 12 months, ranging from 6 to 15 months. LC patients significantly differed, as compared to HS controls, showing lower values in VO_2_ at the peak and at the AT, maximal workload (Watts), O_2_Pulse at the peak, and in OUES values, as well as greater values in VAS dyspnoea (Table 1).

LC had significantly greater values in VE/VCO_2_ slope than HS controls, but they did not differ in BR and PETCO_2_ values (Table 1).

**Figure 1:**
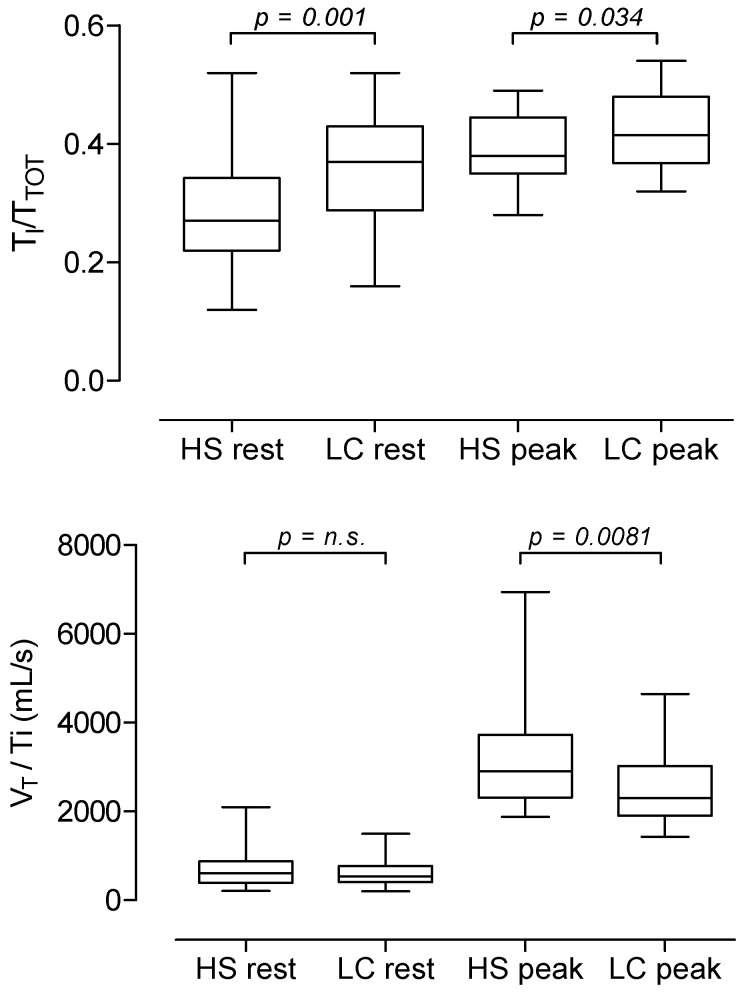
Mean, SD and range values of T_I_/T_TOT_ at rest and at peak of exercise in 40 healthy subjects and 42 Long-COVID patients (*upper panel*) and mean, SD and range values of V_T_/T_I_ at rest and at peak of exercise in 40 healthy subjects and 42 Long-COVID patients (*lower panel*).

**Figure 2:**
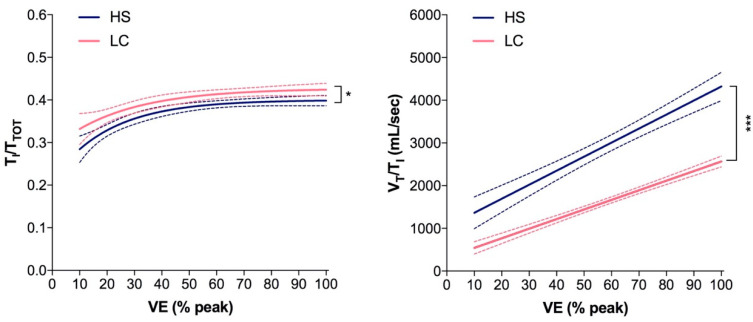
Best fitting curves of data points of T_I_/T_TOT_ (*left panel*) and V_T_/V_I_ (*right panel*) and 95% confidence bands during exercise in 42 Long-COVID patients and 40 healthy subjects. * *p* < 0.05; *** *p* < 0.001.

**Figure 3:**
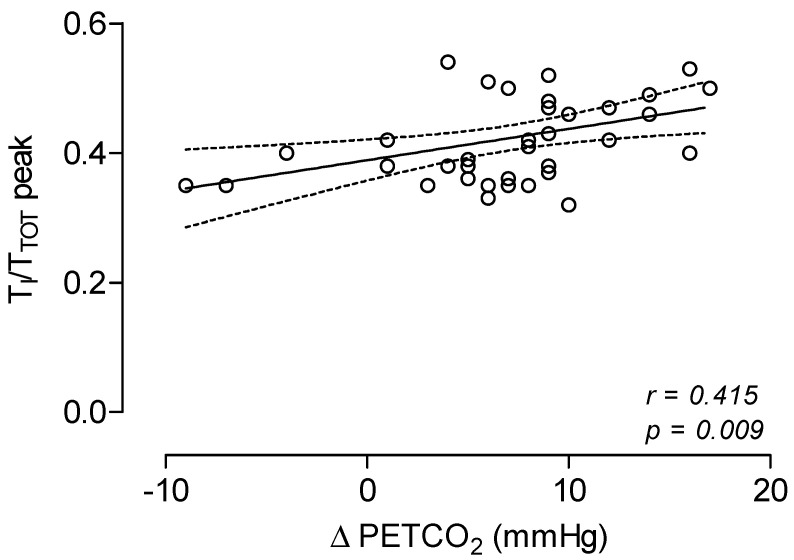
Relationship between T_I_/T_TOT_ values at peak of exercise and Δ PETCO_2_ values in 42 Long-COVID patients.

With respect to the breathing pattern analysis, when compared to HS controls, LC patients did not differ in RR both at rest and at the peak but showed lower values in V_T_ at the peak. LC patients showed significantly higher values of T_I_/T_TOT_ at rest and at the peak of exercise, and lower values in V_T_/V_I_ at peak (Table 1 and Figure 1). The best fitting curves of data points during exercise of T_I_/T_TOT_ and V_T_/V_I_ as plotted to VE (% peak) were significantly different between LC patients and HS controls (Figure 2). In addition, in LC patients, values of T_I_/T_TOT_ at peak were significantly related to ∆PETCO_2_ values (Figure 3).

**Figure 4:**
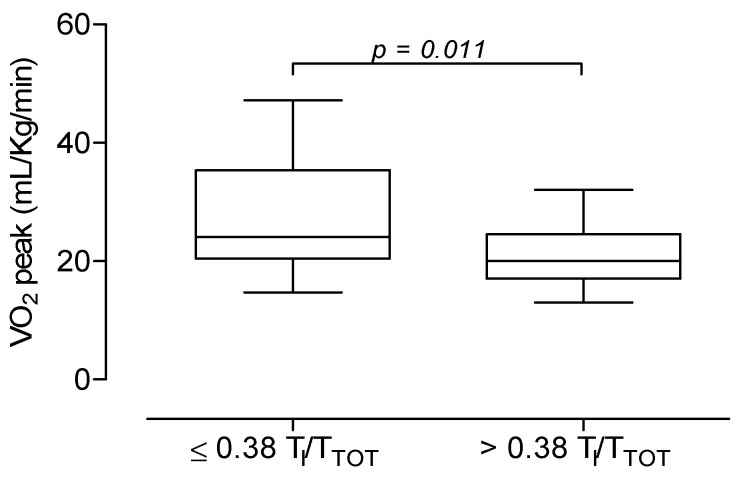

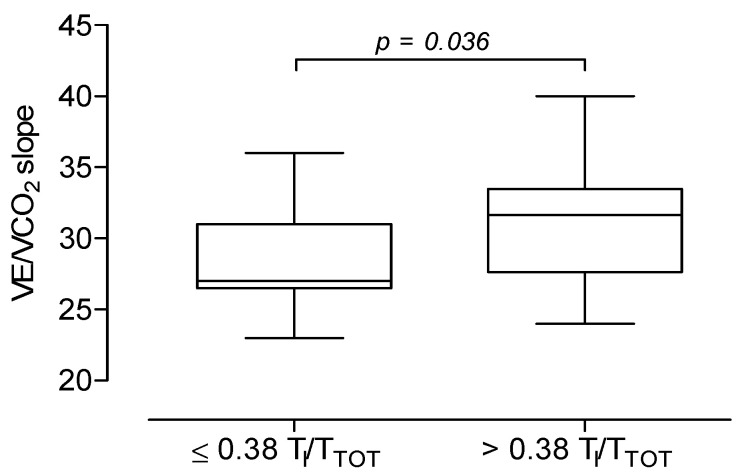
Mean, SD and range values of VO_2_ peak (*upper panel*) and of VE/VCO_2_ slope (*lower panel*) in 13 Long-COVID patients with T_I_/T_TOT_ ≤ 0.38 and in 29 Long-COVID patients with T_I_/T_TOT_ > 0.38 at peak of exercise. Sing et al. [19] also demonstrated a hyperventilatory response during exercise in all patients. Similarly, in a large cohort of patients at approximately three months after the initial diagnosis of SARS-CoV-2 infection, Motiejunaite et al. [5] found an elevated VE/VCO_2_ slope in one third of the study participants, suggesting a high incidence of inadequate ventilation on exertion. In the current study, LC patients had higher values in VE/VCO_2_ slope, as compared to controls, but they did not differ in terms of PETCO_2_, thereby showing ventilatory inefficiency without hyperventilation.

Finally, when LC patients were subdivided according to the T_I_/T_TOT_ cut-off value of 0.38, 29 out 42 LC patients had T_I_/T_TOT_ > 0.38 and showed lower values in VO_2_ peak (21 ± 5 mL/kg/min vs. 27 ± 10 mL/kg/min; *p* = 0.011) (Figure 4) and in maximal workload (114 ± 28 watts vs. 144 ± 60 watts; *p* = 0.035), and greater values in VE/VCO_2_ slope (31 ± 4 L vs. 28 ± 3 L; *p* = 0.036) than the remaining 13 patients with T_I_/T_TOT_ ≤ 0.38.

Importantly, with respect to the ventilatory response, other studies reported significant percentages of LC patients with DB during incremental maximal exercise, with and without hyperventilation [6,7,8]. DB is defined as a neural breathing disorder of the central nervous system, where an abnormal breathing drive results in respiratory discomfort in the absence of underlying cardiopulmonary disease [10]. It is worth of noting that the diagnosis of DB is based on the visual analysis of the plots showing the relationships between V_T_, RR and VE [10]; therefore, the identification of DB is subjective so that comparison of patients with and without DB may not be reproducible.

In the present study, we measured the resting and exertional breathing patterns in a previously rarely-used way, by analysing the T_I_/T_TOT_ and V_T_/T_I_ values in LC patients and in control subjects. Our results agree with the previous ones by Lind and Hesser [12], who studied breathing pattern and lung volumes during maximal exercise in eight young male healthy subjects.

T_I_/T_TOT_ has been called fractional inspiratory time and has been also defined as the duty cycle of the respiratory system, since the level of stress placed on the respiratory muscles is proportional to T_I_/T_TOT_ [11]. Therefore, a prolonged T_I_/T_TOT_ predisposes to respiratory muscle fatigue and is of equal importance to the tension developed by the muscle, as a determinant of diaphragmatic fatigue [20].

During incremental exercise, T_I_/T_TOT_ increases with increasing minute ventilation [12]. Of interest, in the present study we provided the evidence that LC patients, when compared to HS controls, showed higher values of T_I_/T_TOT_ both at rest and at the peak of exercise. In addition, in LC patients the change in PETCO_2_ during exercise was directly related to the duty cycle of the respiratory system.

V_T_/T_I_ has been termed mean inspiratory flow rate and is considered as a measure of respiratory drive, since it was found to be related to indices of respiratory centre output, such as P_0.1_ and the ventilatory response to hypercapnia [21]. During incremental exercise V_T_/T_I_ increases progressively and when related to minute ventilation, the rate of increase in V_T_/T_I_ decreases as minute ventilation rises [12]. In this study, we found that with the increase in exercise and related hyperpnea, LC patients showed an increase in mean inspiratory flow values, lower than that in HS controls, thereby developing minute ventilation at peak exercise was lower than that developed by HS controls.

Overall, our results suggest that LC patients have a breathing pattern that is more prone to diaphragmatic fatigue and less effective than that of the reference controls. Most of the work of the breath is achieved by the diaphragm. After an illness, especially if requiring mechanical ventilation or in conditions of general physical deconditioning, the diaphragmatic movement may be reduced and use of accessory respiratory muscles may occur [22], thereby resulting in an abnormal breathing pattern and breathlessness perception.

The findings of the present study must be interpreted in the context of limitations. The first limitation is due to the lack of breathing pattern data before the SARS-CoV-2 infection, and therefore no comparison before and after infection can be made. Secondly, we did not measure arterial blood gases, and used PETCO_2_ to estimate PaCO_2_ and to exclude hyperventilation syndrome. Furthermore, when using CPET in a non-invasive way, the identification of the primary limitation to exercise can be problematic. However, the present study is, by its nature, a non-invasive study. On the other hand, the strength of this study lies in a well-selected cohort of patients, along with the appropriate group of controls matched for age, gender and BMI. Furthermore, we used an objective approach, based on the measure of T_I_/T_TOT_ and V_T_/T_I_, to investigate the breathing pattern.

In conclusion, we found that patients with previous infection of SARS-CoV-2 who subsequently complained of long-lasting unexplained dyspnoea, showed impairments in resting and on-exertion breathing patterns, along with a CPET profile of deconditioning. Pulmonary rehabilitation, also involving breathing control techniques, might be useful to revert this unpleasant condition.

## Figures and Tables

**Table 1:** Subjects’ characteristics.

Variables	Healthy Controls (No. 40)	Long COVID Patients (No. 42)	*p*
Age (years)	47 ± 11	49 ± 12	0.494
Sex (F/M)	22/18	23/19	0.983
BMI (Kg/m^2^)	25 ± 4	26 ± 3	0.121
FVC (% pred)	108 ± 15	101 ± 17	0.066
FEV_1_ (% pred)	106 ± 12	99 ± 15	**0.025**
FEV_1_/FVC (%)	82 ± 6	81 ± 6	0.255
VO_2_ peak (mL/kg/min)	31 ± 10	23 ± 8	**0.001**
VO_2_ peak (% pred)	105 ± 27	84 ± 21	**0.001**
Workload (Watts)	181 ± 65	123 ± 43	**0.001**
Workload (% pred)	117 ± 36	82 ± 22	**0.001**
AT (mL/kg/min)	21 ± 10	16 ± 8	**0.030**
O_2_ Pulse rest (mL/bpm)	4.9 ± 2.4	4.1 ± 1.3	0.069
O_2_ Pulse peak (mL/bpm)	14.6 ± 4.5	11.7 ± 3.6	**0.002**
OUES (mL/min)	2288 ± 687	1688 ± 686	**0.001**
HR rest (bpm)	75 ± 14	85 ± 15	**0.002**
HR peak (bpm)	152 ± 19	147 ± 15	0.217
HR peak (% pred)	88 ± 9	86 ± 8	0.377
HR recovery (bpm)	25 ± 9	20 ± 10	**0.009**
BR (%)	50 ± 11	51 ± 14	0.768
VE peak (L/min)	72 ± 26	59 ± 21	**0.023**
Vt rest (L)	0.74 ± 0.3	0.74 ± 0.3	0.994
Vt peak (L)	2.37 ± 0.8	1.97 ± 0.5	**0.007**
RR rest (bpm)	14 ±5	14 ± 6	0.770
RR peak (bpm)	31 ± 7	31 ± 9	0.847
PETCO_2_ rest (mmHg)	33 ± 6	34 ± 4	0.319
PETCO_2_ peak (mmHg)	41 ± 5	41 ± 5	0.921
Δ PETCO_2_ (mmHg)	8 ± 6	7 ± 5	0.468
VE/VCO_2_ Slope (L)	26 ± 4	30 ± 4	**0.001**
T_I_/T_Tot_ rest	0.29 ± 0.09	0.36 ± 0.09	**0.001**
T_I_/T_Tot_ peak	0.39 ± 0.05	0.42 ± 0.06	**0.034**
V_T_/T_I_ rest (mL/s)	685 ± 397	605 ± 262	0.286
V_T_/T_I_ peak (mL/s)	3155 ± 1101	2560 ± 850	**0.008**
VAS dyspnoea (mm/watts)	0.48 ± 0.19	0.64 ± 0.22	**0.022**

Values are expressed as mean ± SD. Abbreviations: BMI: Body Mass Index, FVC: Forced Vital Capacity, FEV1: Forced Expiratory Volume at 1st Second, VO_2_: O_2_ uptake, AT: Anaerobic Threshold, OUES: O_2_ Uptake Efficiency Slope, HR: Heart Rate, BR: Breathing Reserve, VE: Minute Ventilation, RR: Respiratory Rate, PETCO_2_: End-Tidal pressure of CO_2_, T_I_: Inspiratory Time, T_TOT_: Tidal Volume duration, V_T_: Tidal Volume, VAS: Visual Analogue Scale. Bold values indicate statistical significance.

## Data Availability

The data are available upon request from the corresponding author.
